# Screening Strategies for High-Yield Chinese Hamster Ovary Cell Clones

**DOI:** 10.3389/fbioe.2022.858478

**Published:** 2022-06-17

**Authors:** Wenwen Yang, Junhe Zhang, Yunxi Xiao, Wenqing Li, Tianyun Wang

**Affiliations:** ^1^ Department of Biochemistry and Molecular Biology, Xinxiang Medical University, Xinxiang, China; ^2^ International Joint Research Laboratory for Recombinant Pharmaceutical Protein Expression System of Henan, Xinxiang, China; ^3^ Institutes of Health Central Plains, Xinxiang Medical University, Xinxiang, China

**Keywords:** Chinese hamster ovary cells, screening system, recombinant therapeutic protein, semi-solid medium, high-yield clone

## Abstract

Chinese hamster ovary (CHO) cells are by far the most commonly used mammalian expression system for recombinant expression of therapeutic proteins in the pharmaceutical industry. The development of high-yield stable cell lines requires processes of transfection, selection, screening and adaptation, among which the screening process requires tremendous time and determines the level of forming highly productive monoclonal cell lines. Therefore, how to achieve productive cell lines is a major question prior to industrial manufacturing. Cell line development (CLD) is one of the most critical steps in the production of recombinant therapeutic proteins. Generation of high-yield cell clones is mainly based on the time-consuming, laborious process of selection and screening. With the increase in recombinant therapeutic proteins expressed by CHO cells, CLD has become a major bottleneck in obtaining cell lines for manufacturing. The basic principles for CLD include preliminary screening for high-yield cell pool, single-cell isolation and improvement of productivity, clonality and stability. With the development of modern analysis and testing technologies, various screening methods have been used for CLD to enhance the selection efficiency of high-yield clonal cells. This review provides a comprehensive overview on preliminary screening methods for high-yield cell pool based on drug selective pressure. Moreover, we focus on high throughput methods for isolating high-yield cell clones and increasing the productivity and stability, as well as new screening strategies used for the biopharmaceutical industry.

## Introduction

Chinese hamster ovary (CHO) cells are one of the most commonly used host cells for the industrial production of recombinant therapeutic protein drugs. Among the top 10 drugs in global sales in 2019, eight are biopharmaceuticals, and seven are monoclonal antibodies produced in CHO cells (Walsh, 2018; Bhutani et al., 2021), mainly due to its ability to express various recombinant proteins with a post translational modification pattern similar to that of the proteins from human cells (Harcum and Lee, 2016). The genome data of CHO cells have a similar proportion of glycosylation-related transcripts to human cells (Xu et al., 2011). The use of CHO cells can also avoid human virus infection and further improve product safety.

The expression of recombinant therapeutic proteins for clinical and commercial production requires the stable integration of gene of interest (GOI) into the CHO genome. The most common approach is to randomly integrate GOI into the host genome as part of a plasmid and then screen transgenic cells (Noh et al., 2019). The homogenous levels of protein expression between individual transfected cells are rarely observed due to factors such as cell-to-cell heterogeneity, difference in gene copy number and chromosomal environment (West and Fraser, 2005; Lee et al., 2019). The heterogeneity caused by random gene integration can be alleviated by single-cell sorting, which contributes to the consistency of product quality and manufacturing. Another reason for single-cell sorting preference is that after transfection and selection, high-yield clones appear infrequently in heterogeneous cell populations (Lattenmayer et al., 2007; Chusainow et al., 2009).

In addition to the unpredictable effects of random integration, CHO cell lines and most immortalized cell lines are subjected to highly clonal variation in genotypes and phenotypes (Pilbrough et al., 2009; Lewis et al., 2013). This trait is not desirable in industrial production because it complicates the screening of sufficient clones to avoid heterogeneity. Given that the expression of target protein consumes energy and resources from the host, the growth of low-yield cell lines with different expression levels is generally faster than that of high-yield cell lines, further leading to a gradual decrease in their expression level (Zeyda et al., 1999). Isolation and expanding individual high-yield cells into a population of highly expressing cells with clonal properties are necessary. Screening can take advantage of modern technology and robotics; however, the development of cell line production is still a time-, labor-, and capital-intensive effort that typically takes 6–12 months. Clonal screening and selection involve many analytical screening techniques to ensure the selection of high-yield clones that produce recombinant proteins with high titers, good quality, and stability without productivity loss over time (Ritter et al., 2017).

Therefore, advances in cell line development (CLD) technology are critical to support the rapid development of recombinant protein products. Improvements in development processes and the ease of producing high-yield cell lines in research conditions contribute to the rapid advancement of biosimilars and innovative products. In the production of innovative recombinant therapeutic protein drugs, shortening the time to market is also beneficial for biopharmaceutical manufacturers to maximize the profitability of the biologic product during the limited patent exclusivity period. Advances in CLD technology focus on the improvement of protein expression and screening technologies for high-yield clones. In this review, the advances in high-yield cell clone screening and evaluation techniques in cell clone sorting are summarized and assessed for the industrial production of therapeutic protein drugs.

## Cell Pool Selection Markers and Screening Methods

### Screening Systems Based on Metabolic Pathways

The main challenge in CLD for recombinant protein production is to generate and isolate rare high-yield clones in a short period of time from thousands of low-yield or unstable clones. The two most commonly used expression systems are based on metabolic pathway screening methods to establish stable, high-yield recombinant CHO cell lines: dihydrofolate reductase (DHFR) system and glutamine synthetase (GS) system (Table 1). DHFR catalyzes the conversion of folic acid to tetrahydrofolate, a process required for the biosynthetic pathway that produces glycine, purine, and thymidylate (GHT). The DHFR system can be used in CHO cell mutant strains, such as DXB11 and DG44, in which the DHFR gene is mutated or deleted. The growth of such nutrient-deficient cell lines requires a medium containing GHT or transfection of DHFR. In the DHFR system, GOI is generally transfected into host cells with DHFR gene in the same expression vector.

**TABLE 1:** Common selection markers used in cell line development.

Selection markers	Screening reagents	Selection principle	Concentration range
DHFR	MTX	A folic acid antagonist that causes cytotoxicity by inhibiting DHFR activity and thus nucleic acid synthesis	25–1000 nM
GS	MSX	Inhibits glutamine synthetase gene	25–500 μM
Puromycin acetyltransferase	Puromycin	Aminoglycoside antibiotics that block protein synthesis in mammalian cells by interfering with ribosome function	10–50 μg/ml
Blasticidin deaminase	Blasticidin	A nucleoside antibiotic that specifically inhibits protein synthesis in prokaryotes and eukaryotes by interfering with the formation of peptide bonds in ribosomes	5–50 μg/ml
Aminoglycoside phosphotransferase	Geneticin	Aminoglycoside antibiotics, one of the most commonly used resistance screening agents for stable transfection	200–700 μg/ml

**FIGURE 1:**
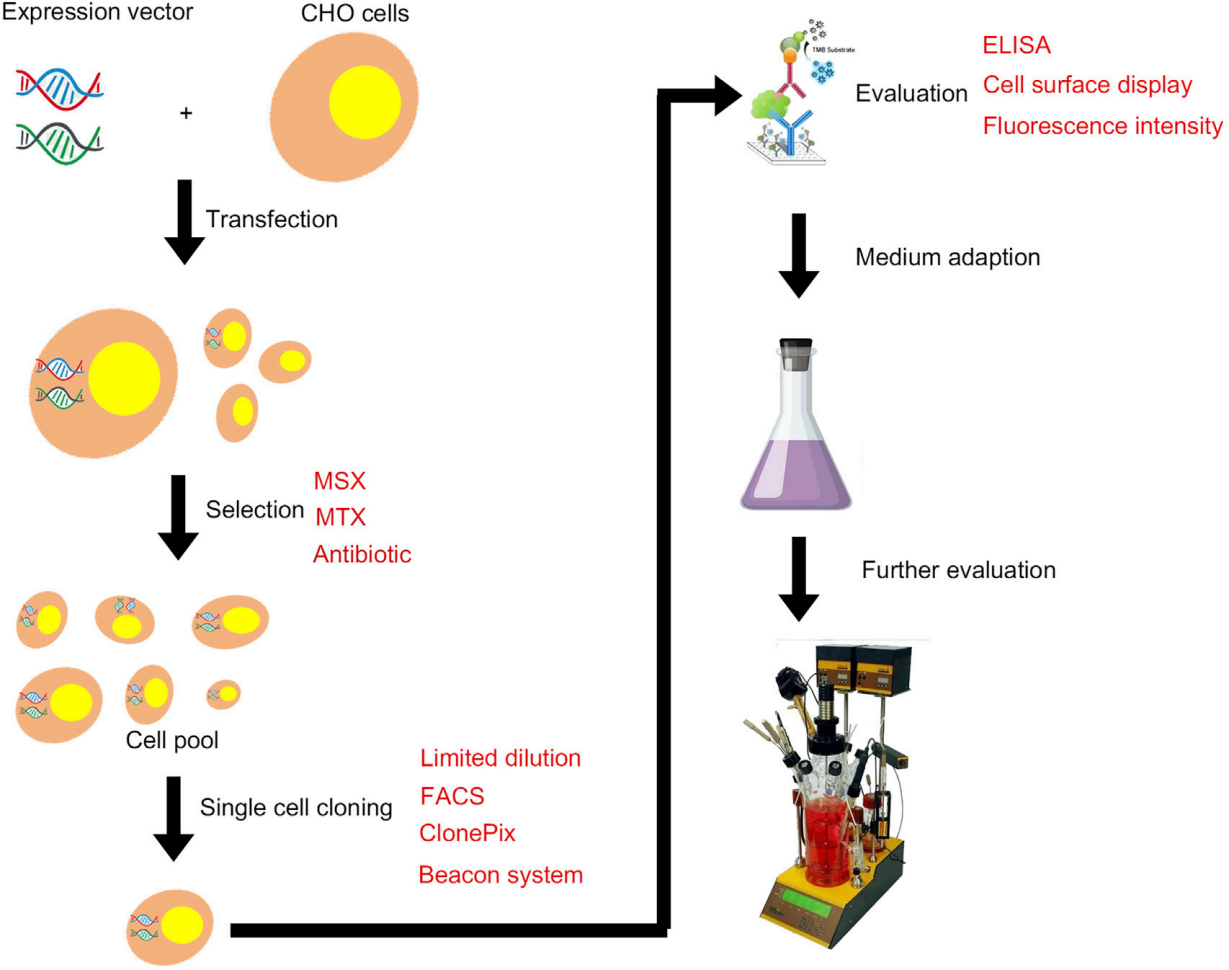
Illustration of the typical development of a mammalian cell line for recombinant protein manufacturing.

The transfected cells are cultured in the medium without GHT, and the surviving cell pool contains GOI and DHFR genes in their genomes. When the cell medium contains methotrexate (MTX) (Figure 1), the dihydrofolate reductase is inhibited, and the gene is amplified through feedback regulation. All genes in the upstream and downstream range of 100–1,000 kb are amplified accordingly (Urlaub et al., 1986). Therefore, GOI can be amplified by inserting within the range of this site. The DHFR system is widely used because of its high efficiency in gene amplification. The first bottleneck in isolating high-yield cell lines is the selection of clones with the best productivity and growth rates from the amplified cell pool. These characteristics are partly dependent on the copy number. Standard methods include isolating individual clones by limiting dilution and cloning cylinders (De et al., 2004; Quiroz and Tsao, 2016; Zhou and Shaw, 2018). Assessment of the growth rate of each clone and the productivity of target protein revealed that the process is time-consuming and thus hinders the development of new biopharmaceuticals. Two different strategies can be adopted for selecting high-yield clones. The first approach involves isolating individual clones from the first concentration level of MTX selection, then placing each clone in a relatively high concentration level of MTX selection, and finally isolating the individual clones again. Subcloning can also be performed to optimize homogeneity (Kim et al., 1998). The second strategy involves pooling clones at each stage of MTX selection and isolating single clones from the final MTX resistance library. A study compared the effectiveness of the two strategies by examining the antibody productivity of 30 parent clones and 10 parent cell pools after undergoing MTX amplification program (Jun et al., 2005). High-yield clones were isolated from the cell pool at an antibody titer of 5 μg/ml within 15 weeks. After approximately 17 weeks, high-yield cell clone strains were isolated from 30 parent clones, with the highest subclone reaching a titer of 17 μg/ml. The individual cloning strategy proved to be labor intensive and time consuming due to the additional cloning steps, and the scheme was not improved by incorporating MTX in the initial selection of transfectants. The cell-pool strategy is less labor intensive, but the highest producers are approximately one third of those isolated using the individual cloning strategy. Therefore, a selection strategy based on individual clones is favored for establishment of high-producing CHO clones because it is more efficient to perform cell cloning at the initial selection stage of parental cell clones (Imanaka and Aiba, 1981). The first-round pool selection at the outset followed by LDC and the pool selection using higher concentration MTX might give higher titers (Noh et al., 2013).

**FIGURE 2:**
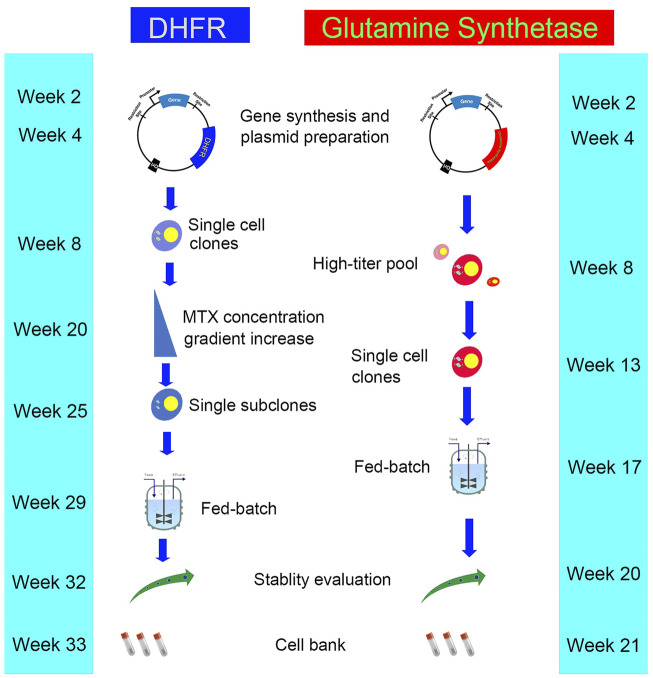
The workflow and timeline for DHFR and GS system.

The GS system utilizes the GS gene as a selection marker. GS synthesizes glutamine from ammonia and glutamic acid in cells, which is then hydrolyzed by adenosine triphosphate (ATP) to provide energy. L-methionine sulfoximine (MSX), a GS inhibitor, was added into a culture medium without exogenous glutamine (Figure 1). The results showed that the GS gene and its associated target genes were amplified effectively, thus improving the expression level of target genes. The advantages of this system are as follows. 1) The CHO-K1 cell line with genetic defect is not needed as the host cell, while GS-knockout cell line is a better expression platform. 2) CHO-K1 cells are easy to culture and grow faster. 3) Glutamine need not be added in the culture medium, thus avoiding the problem of high ammonia level in the culture system caused by glutamine decomposition, reducing the difficulty of process control, improving cell fermentation density, and prolonging cell survival time. Wild-type CHO cells have endogenous GS genes that can be selected by adding MSX at low levels (Bebbington et al., 1992; Brown et al., 1992). CHO-K1 cell line was first used in the GS system (Cockett et al., 1990). GS-knockout cell lines are also developed to improve the efficiency of cell line screening, and the use of the GS-knockout CHO host cell line facilitates the rapid generation of high producing clones (Fan et al., 2012; Noh et al., 2018). In recent years, the genome editing tools including Clustered Regularly Interspaced Short Palindromic Repeat (CRISPR)/CRISPR-associated protein 9 (Cas9) and zinc finger nucleases (ZFNs) have been used to generate GS-knockout cell lines with desired growth and recombinant protein expression characteristics (Grav et al., 2017; Feary et al., 2021; Huhn et al., 2021). The DHFR system requires a long time for gene amplification through the gradual increase in MTX, whereas the GS system can achieve sufficient expression levels through a round of selection and amplification, thus reduce the total time required for cell line generation (Barnes et al., 2000). The workflows and timelines for DHFR and GS system are shown in Figure 2. In addition, the GS system diminishes the accumulation of ammonia in the medium because overexpressed GS catalyzes the conversion of glutamic acid and ammonia to glutamine (Wurm, 2004).

The high expression of GS system depends on the existence of MSX to a certain extent, but MSX is not conducive to industrial production. Mutants with low activity were obtained through GS modification. Hence, the CHO cell lines with low activity of GS as the screening marker could efficiently express antibodies after MSX removal (Lin et al., 2019). As an attenuated selective marker produced by the CHO cell line, GS mutation R324C can substantially increase the antibody production in the stable transfection pool and obtain stable clones with high antibody productivity (Lin et al., 2019). In addition to DHFR and GS systems, other screening systems based on defects in different metabolic pathways are currently being developed. In CHO cell lines lacking the proline metabolic pathway function, the pyrrolin-5-carboxylate synthase gene is applied as a selection marker to allow the use of proline-free medium for selection (Sun et al., 2020). CRISPR-Cas9 is employed to knock out the genes encoding the last two steps of a bifunctional enzyme that catalyzes the *de novo* synthesis of pyrimidines and purines (5-aminimidazolium-4-formamide ribosylnucleotide transferase/IMP cyclic hydrolase [ATIC], respectively). The survival of these bistrotrophic cells depends on the availability of purine and pyrimidine sources or the transfection and integration of the open reading frame that encodes these two enzymes. One of these double trophic deficiency forms is used to select a stable transfector carrying the target protein. The transfected clones could stably produce large quantities of recombinant proteins. This double trophic deficiency provides a rapid and effective selection method for the separate or simultaneous transfer of multiple target genes into CHO cells by using readily available commercial mediums without purine and pyrimidine (Zhang et al., 2020). Polyamine plays an important role in cell proliferation, DNA replication, transcription, and translation (Igarashi and Kashiwagi, 2021). Depletion of putrescine and spermine in cells resulting in the stagnation of cell growth and ultimately death. The CHO-K1 cells of a serum-free medium might require supplementation of putrescine because these cells cannot facilitate the polyamine production of the first kind of enzyme, arginase, based on their phenotype. A proline-based selection system was also constructed and could obtain stable and high-yield cell lines by screening cells in the medium without L-ornithine and putrescine (Sun et al., 2020).

### Screening Systems Based on Exogenous Resistance Genes

Another selection method is the use of antibiotic-based resistance genetic markers that confer resistance to antibiotics, such as Geneticin, Zeocin, Hygromycin B, and Puromycin (Capecchi, 1989) (Table 1; Figure 1). Resistance gene screening is usually combined with traditional limited dilution method to obtain monoclones, and high-yield clones are further identified by ELISA and Western blot. The most common traditional method for screening monoclones is the limited dilution method, which requires multiple rounds of subclones to ensure monoclonal properties. Given that the newly transfected cells are highly heterogeneous, the cell density must be increased in the first step to maximize the number of cells within a single wells. Screening is usually performed in a 96- or 384-well plate. In the first screening step of subclone, the newly transfected cells undergo limited dilution at an average cell density of 2,500 cells during initial plating (Borth et al., 2000). After a period of pressure screening, the surviving cells grow and form a cell population. A single cell population in the well is called monoclonal cell. If the formation of monoclonal cells in the first round is insufficient, then a second round subclone is selected from the cells of the selected monoclonal in the first round. This step remarkably reduces the heterogeneity of cells in the second round. A stable monoclonal cell could then be obtained after a period of pressure screening with a low plating density in the second round. Although limited dilution can be used as an effective method, its several limitations prevent it from being a viable screening technique for high-yield clones. First, this technique is a time-consuming and labor-intensive process with a long screening cycle. For cell lines expressing non-secreted proteins, the cells are usually permeabilized or disrupted, ELISA can be performed, and downstream experiments are required to determine the productivity of each clone cell. The whole process can take up to 8 months due to the considerable additional work required to fully characterize each monoclonal cell line.

In recent years, the method of antibiotic resistance genes has been successfully optimized for the selection of high-yield cell lines. One approach is to weaken antibiotic resistance genes on plasmids containing target genes (Bandaranayake and Almo, 2014), thereby allowing the selection of high-yield cell lines at low doses of selective antibiotics and avoiding the problem of slow cell growth that occurs in the presence of high antibiotic doses (Sautter and Enenkel, 2005). Weak promoters, such as herpes simplex virus (HSV) promoter, had been applied to achieve this purpose (Niwa et al., 1991). Another approach is to associate the target gene with the resistance gene through polycistronic elements, such as the most commonly used internal ribosome entry site (IRES) and 2A elements, to minimize non-expressed clones and achieve stable monoclonal antibody productivity without gene amplification (Ho et al., 2012; Ho et al., 2013).

In addition, different antibiotic resistance genes have various selection effects. Lanza et al. (2013) identified the order of bleomycin > hypopycin B > neomycin > puromycin by analyzing the expression of green fluorescent protein (GFP) in two commonly used human cell lines. Although these simple methods are useful and the materials are easily obtained, antibiotic selection could reduce the rate of cell growth and even result in morphological changes. Only a few reports comprehensively compared the efficiency and effect of different selection systems in CHO cells (Lanza et al., 2013; Yeo et al., 2017). More advanced selection methods should be developed to obtain additional high-yield cell lines.

## Screening Methods of High-Yield Monoclonal Cells

### Screening Methods Based on Fluorescence Signals

Flow cytometry has rapidly become one of the most widely used tools to select cells with desirable phenotypes. In particular, fluorescence-activated cell sorting (FACS) classifies cells based on the determined fluorescence level (Figure 1). One of the most desirable features of flow cytometry is its high throughput capability to analyze millions of cells per minute, thus saving time, manpower, and money/resources (Kumar and Borth, 2012). The sorting rate is determined by multiple factors, such as the size and density of cells. In addition to being a tool for selecting highly expressed variants, flow cytometry can also be used to analyze cell growth and metabolism, both of which can affect cell productivity (Hinterkörner et al., 2007).

The expression level of the target protein must be characterized by fluorescence signals to successfully utilize flow cytometry. The available methods are generally divided into two categories: 1) GOI co-expression with reporter gene whose expression levels in different clones can be used to determine GOI expression levels, and 2) detection of secreted GOI on or near the surface of a single producing cell by fluorescently labeled antibodies specific to GOI. Reporter-gene expression is usually maintained at a lower level than GOI expression to reduce the burden of reporter-gene expression on cells. This condition can be achieved by using a defective promoter, a weak Kozak sequence, or by placing internal IRES between the reporter gene and GOI (Cha and Bentley, 2007). Common reporter genes include green fluorescent protein (GFP), yellow fluorescent protein (YFP), and red fluorescent protein (RFP) (Sleiman et al., 2010). GFP is toxic to some cells at high concentrations and therefore reduces the growth and stability of generative cells (Zeyda et al., 1999). Cell productivity is positively correlated with the fluorescence intensity of GFPS65T, an eGFP mutant. Compared with continuous MTX selection, three rounds of separation (sorting is 2 weeks after each round of growth) produce more than six times the productivity of cloning (Meng et al., 2000). Although the time required is the same for artificial and cell sorting, the workload is greatly reduced because ELISA is not required for high-yield cell strain selection. In addition, further selection pressure can be applied in combination with cell sorting. This finding has been verified in CHO cells that co-express the metallothionin–green fluorescent protein fusion protein and target protein; due to the combination of metallothionin-based gene amplification with FACS, high-yield cell strains can be isolated within 4 weeks (Bailey et al., 2002).

Most biological drugs are monoclonal antibodies and have heterotetramer structures composed of isomolar light and heavy chain polypeptides. Hence, the efficiency of antibody assembly is largely dependent on the expression ratio of these chains. The ratio of heavy to light chains affects the final antibody production titer. Therefore, selecting the cell lines with the optimal ratio of heavy to light chains is crucial for monoclonal antibody assembly (Schlatter et al., 2005). FACS assay based on heavy- and light-chain assemblies provided insights into the optimal antibody expression in CHO cells by first performing a two-color sorting of green fluorescent protein and yellow fluorescent genes that fused with recombinant antibody heavy- and light-chain genes, respectively (Sleiman et al., 2010). Fluorescent fusion antibody chains are co-expressed by IRES-based vectors. Dual fluorescent clones selected by FACS showed a 38-fold increase in antibody production within 12 weeks relative to that of their parent pool.

Another example of a fluoresce-based automated system technology is the combination of cell growth in semi-solid medium with automated fluorescence detection and screening by automated cell pickers, such as clonal fluorescence microscopy (Clonepix). Roy et al. (2017) developed a method to analyze the expression levels of individual immobilized cells by growing cells in semi-solid medium, providing the nutrients necessary for cell growth, and adding fluorescently labeled antibodies to the surface of the semi-solid medium. The cells must be characterized by flow cytometry in the early cloning selection stage to identify cell lines with high productivity potential and help eliminate unstable cell lines. The unique combination of clonographic fluorescence screening and flow cytometry methods contributes to the efficient isolation of clone cell lines at high productivity within 15 weeks and their possible application to NS0 and CHO cells. One of the greatest advantages of semi-solid medium technology is that high-yield clones can be isolated using an automated cell selector after productivity analysis by an imaging system. Hence, the time and labor required to select high-expressing variants are reduced, and the selection of high-yield cells is better than that in traditional manual methods. Compared with sorting via flow cytometry, the main advantage of this method is that the resulting fluorescent signal is an integral part of the productivity during clone development. In addition, flow cytometry is an indirect measure of the secretory rate during clone sorting.

The Clonepix system detects fluorescence by adding fluorescence-bound “clone detection” reagents (Figure 1). Hou et al. (2014) used the CloneTable to develop stable, highly expressed clones with specific productivity exceed 20 pg/cell/day (pcd) after 4 weeks. CloneTable evaluates each clone based on user-defined parameters such as size, shape, fluorescence, and position. Among the 384 clones selected from 96-well plates, 104 were identified as having industry-relevant estimated productivity, namely, higher than 20 pcd. This system (such as tables and cloning cells based on fluorescence accelerometer) is provided by the automation, and the cells can grow for 2–3 weeks in the semi-solid medium without the fact of subculture. As a result, the number of clones to be filtered is increased, and the amount is lower than that in the traditional artificial selection.

### Screening Methods Based on Site-Specific Integration

The influence and heterogeneity of random integration sites of target genes on protein expression is one of the important reasons for increasing the workload of screening. Target gene integration has the potential to reduce clonal variation and thus accelerate the selection of high-yield CHO cell lines (Kelley, 2020; Sun et al., 2020). Integrating target genes into preselected genomic sites enables the predictable generation of isogenic cell lines with consistent phenotypes and expression level (Grav et al., 2018). After site-directed integration in mammalian cells was reported in the late 1980s (Thomas and Capecchi, 1987) and early 1990s (O’Gorman et al., 1991; Schlake and Bode, 1994), several methods for targeting gene integration have been developed. These methods utilized site-specific recombinases such as CRE, FLP, and BXB1 for site recombination, Recombinase mediated cassette exchange (RMCE), or programmable nucleases such as CRISPR/Cas9 (Wirth et al., 2007; Turan et al., 2013; Hamaker and Lee, 2018). However, this strategy has not been used on an industrial scale largely due to the low specific productivity of the target protein resulting from the single-copy GOI integration and the challenge of finding highly active transcription sites (hot spots) in the genome (Baumann et al., 2017; Hamaker and Lee, 2018).

Several companies have published reports of using the RMCE system to produce monoclonal antibodies. In 2013, Genentech established a CRE based monoclonal antibody expression platform for RMCE by screening transcriptional active sites in the CHO genome (Crawford et al., 2013). Inserting a single expression cassette with heavy and light chain genes can produce stable cell lines for five different mAbs expression levels with a repeatable qP of 3–4 pcd. Inserting two monoclonal antibody expression cassettes into the same genomic site doubles the specific productivity to a maximum of 10 pcd. Pfizer developed FLP-based RMCE and BXB1-based RMCE platforms for the expression of single-copy monoclonal antibodies to the qP of 3 pcd (Zhang et al., 2015; Inniss et al., 2017). In 2020, Genentech showed that in the RMCE system, monoclonal antibodies can be expressed at a qP of 20–50 pcd by integrating multiple copies of heavy and light chains into a single genomic site (Carver et al., 2020). qP can be improved by the site-specific integration of multiple copies of the target gene at multiple high transcriptional activity site (Sergeeva et al., 2020). With the identification of many highly transcriptional active sites, site-specific integration may be the most effective and rapid method for the selection of high-yield clone cell lines.

### Label-Free Screening Methods Based on Cell Surface Display Technology

Screening methods using resistance genes or report gene negatively affect the host cell metabolism. The introduced gene, other than the target gene, will occupy the cell protein synthesis resources, resulting in the decrease in target protein expression. With the progress on instrumental analysis methods, many high-yield clonal screening methods without labels have been established.

By combining surface plasma resonance imaging (SPRI) and self-sorting micropore technology, Abali et al. (2017) realized the real-time monitoring, tracking, and quantification of antibody secretion of a single cell without labeling and separated the selected cells by pressing cells out of micropores using the micropore position coordinates obtained by SPRI. The target cells can be removed aseptically from the array of micropores for further culturing. After overnight culturing, thousands of cells can be screened out in hours rather than weeks.

Chakrabarti et al. (2019) used a simple living cell staining method to detect mitochondrial membrane potential, a key indicator of cell metabolic activity, for the identification of cells with high productivity in the FACS step. The intensity of the burst fluorescence signal is related to the titer of batch culturing of producing clones, and high-yield clones are selectively enriched via the cell sorting based on the optimal ψM staining strength from the non-monoclonal cell pool. These clones are phenotypically stable for the production of recombinant proteins.

In addition to establishing stable CHO cell lines that produce therapeutic recombinant proteins by antibiotic and/or metabolic selection, Muralidharan–Chari et al. (2021) reported a new technique, namely, PT Select, which utilizes siRNA to clone upstream of GOI and generate functional PT Select-siRNAs through ligation to achieve cell aggregation. Cells with stable integration of GOI are selected and isolated from the cells without GOI by transfecting CD4/siRNA gene regulated by PT Select-siRNAs and using the variable expression of CD4 on the cell surface. PT Select quickly establishes a cell pool with the same stability and productivity as the pool generated by traditional methods. This pool can be further used to monitor productivity changes caused by clonal heterogeneity and identify single low-yield cells.

Traditional static batch culturing screening is not related to suspension batch culture used in production and therefore has minimal predictive utility. Small batch screening of feedstock in suspension culture is associated with the bioreactor process, but the number of clones that can be manually screened is limited. When combined with automated liquid handling, small culture systems, such as shaken deep-well plates, offer an effective way to screen many clones. Wang et al. (2018) developed a deep-well plate culture platform with a shaking table to effectively screen 384 clones using the suspension fed-batch method. The set-up was equipped with an automated liquid handling system that integrates cell counting and protein titer measurement instruments. Stirring speed and culture volume are the key factors that correlate deep suspension culture with flask culture. With this automated system, the number of clones to be screened is five times more than using manual batch flask or flask culture tube. Statistical analysis in this study showed that 384 is the optimal number of clones for screening, with a 99% probability that six clones in the 95th productive percentile are included in the screening process (Wang et al., 2018). Cell lines with production levels greater than 6 g/L can also be identified.

Although most RNAs are spliced into transcripts that encode secretory proteins, Aebischer-Gumy et al., 2020 described a mammalian expression construct (SPLICELECT™) that allows a portion of secretory proteins to be redirected to the cell surface by using selective splicing. However, a weakly spliced donor site produces a secondary transcript that encodes another C-terminal transmembrane region. In their study, the cell surface in stable cell lines is correlated with the levels of target secreted proteins and heterodimer in the case of bispecific antibodies to regulate the level of cell surface display and secretion in an independent manner. In addition, the constructed antibody could be used for the rapid screening of multiple antibody candidates in the transient post-transfection binding test. On the basis of the correlation between product quantity and quality of secretory and membrane display products and the flexibility of the constructed plasmid in terms of cell surface display/secretion levels, SPLICELECT is a valuable tool with many potential applications including industrial cell line development and antibody engineering.

## Other Screening Methods

Novel cell line screening methods mostly rely on high-throughput technology to improve the screening efficiency. Moreover, several high throughput methods for CLD are listed, and Tejwani et al. (2021) give more details in the development of these technologies.

### Cyto-Mine

In recent years, with the development of microfluidic technology, the single cell separation by water-in-oil has become more and more mature. It provides a completely different innovative technology platform for improving the traditional method of separating single cells. The sphere fluidics produced by Cyto-Mine^®^ is a high-throughput microfluidic single-cell analysis and screening system, which uses the droplet wrapping technology to quickly separate and wrap thousands of single cells in a short time to form hundreds of skin liters of small droplets, making each droplet an independent system for cell culture and detection of single cells (Josephides et al., 2020). By detecting the expression and secretion level of single cells, the cells with the highest antibody expression level can be quickly detected and screened within a few hours, and then sorted into a separate well in the culture plate. In the whole process, the cells are wrapped in droplets to avoid the influence of shear force from the fluid, which ensures a large number of valuable single cells are easier to form monoclonal antibodies. In addition, the Cyto-Mine platform also integrates high-speed imaging technology to collect images of droplets flowing in microfluidic pipes. By analyzing the number of cells in the droplet, it can automatically provide evidence for screening the origin of monoclonal cells. The above processes are integrated into one system, and the whole process only takes a few hours. It can easily complete 1∼2 rounds of screening processes in one working day, thus greatly saving time and cost for pharmaceutical discovery and production.

### OSCAR

New technologies such as the OSCAR expression system have emerged to provide faster development of high-yield stable cell lines and reduce cost than traditional systems for accelerated commercialization (Costa et al., 2012). Evaluation on the monoclonal antibody in CHO-K1 cell line transfected with OSCAR revealed that this technology is relatively quick, simple, and has no negative effect on cell growth characteristics. However, the value of this approach in the biopharmaceutical industry remains to be explored.

### Verified *In-Situ* Plate Seeding

Verified *In-Situ* Plate Seeding (VIPS) was developed by Solentim and is combined with Cytena single cell printer instruments, which combine cell seeding with microscopic imaging to ensure the single cell deposition and origin of derived clones (Yim and Shaw, 2018; Pekle et al., 2019). Compared with the limiting dilution single-cell cloning workflow, this strategy dramatically reduces the number of microtiter plates needed for the single-cell cloning of industrial cell lines by combining single-cell printing and plate imaging with manual image verification. Therefore, the number of acquired and stored high-resolution images is reduced.

### Beacon Platform

The Beacon platform from Berkeley Lights utilizes nanofluid and opto-electropositioning technology for the culturing, manipulation, and characterization of thousands of cells in parallel on a single chip via software-controlled operations (Le et al., 2018; Le et al., 2020; Tihanyi and Nyitray, 2020). Compared with the FACS-enabled microtiter plate-based workflow, the Beacon platform could generate comparable clonal cell lines with reduced resources. Recently, Opto Cell Line Development 2.0 was developed by Berkeley Lights. It is used to screen and select clones whose titer is 1.5–3 times higher than that selected by traditional clone selection technology. From the moment the cells are cloned into the NanoPen^™^ chamber, they can be intuitively tracked through on-chip culture, measurement and clone recovery for many days. The top level clones can be recovered with more than 99% monoclonal assurance and an advanced packet containing a separate visual record of all clones.

## Conclusion and Future Perspectives

In recent years, the progress in the field of life science has continuously optimized the process of CLD. The key steps to optimize the integrated CLD process are as follows: Set the workflow and then use gene amplification methods for clonal selection to select cell lines suitable for this workflow. The synthetic vector technology is used to improve the expression level of recombinant proteins. Although the selection of high-yield clones remains a challenging task, the choice of optimal cell lines has been simplified using more advanced techniques. This selection process is important in the continuous journey of unraveling the cellular mechanisms necessary to achieve high-quality protein production. In the future, more innovative screening methods will be explored and developed.

It is worth noting that there is no one particular method suitable for all cases. Each stage of the CLD workflow needs to be optimized for a specific clone. The performance of cell lines in large-scale bioreactors is another important consideration in their application for the industrial production of recombinant protein drugs. However, with the establishment of automatic cell biology platform, the corresponding process can be optimized step by step. This is also an effective way to reduce labor-intensive processes, cost and risks. Yet, combining these automatic models will help broaden our understanding and improve the various screening systems used for the biopharmaceutical industry. These powerful tools will bring valuable contribution to the advance of high-yield cell clone screening and development in biotechnology.
